# Editorial: Protection and healing in the digestive system and other tissues: Novel factors, mechanisms, and pharmaceutical targets

**DOI:** 10.3389/fphar.2022.1116643

**Published:** 2022-12-20

**Authors:** Thomas Brzozowski, Predrag Sikiric, Duan Chen, Ki-Baik Hahm, Sven Seiwerth

**Affiliations:** ^1^ Jagiellonian University Medical College, Faculty of Medicine, Cracow, Poland; ^2^ School of Medicine, University of Zagreb, Zagreb, Croatia; ^3^ Norwegian University of Science and Technology, Trondheim, Norway; ^4^ Digestive Disease Center, CHA Bundang Medical Center, CHA University, Seongnam, South Korea

**Keywords:** gastrointestinal injury, protection, ulcer healing, pharmacotherapy, cancer, drug repurposing, non-digestive tissues

Damage to the surface layer of the gastrointestinal tract, caused by strong irritants, necrotizing agents or drugs such as non-steroidal anti-inflammatory drugs, is a serious clinical entity that can ultimately lead to the development of a chronic stage, including peptic ulcer disease and, consequently, neoplastic changes, which requires greater effort from the side of pharmacotherapy and even surgery. The original term “cytoprotection” was pioneered by Andre Robert who discovered the protection of the gastric mucosa by endogenous prostaglandins (PGs) stimulated by intragastric administration of “mild irritants” or by exogenous PGs used at doses that induced this protective effect when administered in non-antisecretory doses. Later, most researchers focused on the mechanism and pharmacotherapy of gastrointestinal injuries and chronic gastroduodenal ulcers, more relevant to clinical gastroenterology. Both protection and healing are universal and can affect other then digestive tissues and even body organs nowadays defined as “organoprotection.” Therefore, our current Research Topic was dedicated to the latest developments in the field of injuries, protection and healing not only of gastrointestinal organs but also non-digestive cells and tissues. We have encouraged world-recognized experts working in that field to address this Research Topic focusing on understanding of mechanism of action of central and peripheral molecular regulators and pharmacological agents that provide new insights into the concept of injury, protection, and healing.

The articles received by the journal were carefully reviewed, thus offering a high-quality Research Topic. As editors of this Research Topic, we have thoroughly enjoyed serving as Guest Editors and reviewing a wide variety of interesting manuscripts related to new targets and therapies to protect and heal gastrointestinal and non-gastrointestinal cells, tissues, and organs, including the utility of new drugs, drugs repurposing, pharmacological tools, and an introduction of original approaches. Below we summarize the main findings and perspectives detailed in each of the twenty-seven accepted articles.

An important report by Zhou et al. revealed that NAFLD is exacerbated by insulin in T2DM and treatment with liraglutide, a GLP-1 agonist, ameliorated hepatic steatosis and liver damage by directly activating hepatic GLP-1R to stimulate PPARα expression by activating the PKA-AMPK signaling pathway and reducing ROS-related apoptosis. These results support the notion that liraglutide may be useful in the treatment of T2DM. Rabben et al. studied the chemopreventive effect of isocyanates administered before tumor initiation in mouse models of gastric cancer, including INS-GAS mice, against potentiating the efficacy of cytotoxic drugs such as cisplatin, prompting the interest of clinicians to use a combination strategy in gastric cancer. Given the side effects such as drug resistance and failure of conventional 5-ASA drug therapy for inflammatory bowel disease, He et al. demonstrated a synergistic effect of 5-ASA and vorinostat (SAHA), a histone deacetylase (HDAC) inhibitor, in the treatment of experimental dextran sulfate colitis *in vivo*, associated with lower toxicity and p65 mRNA expression in Caco-2 and HCT-116 human colonic epithelial cell lines *in vitro*. These findings highlight the efficacy of SAHA acting synergistically with 5-ASA in the treatment of human UC, primarily by inhibiting the nuclear factor kappa B (NF-κB) signaling pathway. Rabben et al. used computational predictions based on the gene expression profiles of the human and mouse gastric cancer (GC) model to reposition ivermectin in the treatment of GC, validated by *in silico*, *in vitro* and *in vivo* methods. Ivermectin reduced the tumor size which was associated with inactivation of WNT/β-catenin signaling and cell proliferation pathways and activation of cell death signaling pathways. In their review, Seiwerth et al. have demonstrated the summary of practical applicability the pleiotropic drug BPC 157 capable of curing the cutaneous and other tissue wounds in rodent models by preventing vessel constriction, the formation of fibrin mesh which may stabilize the platelet plug, thus promoting resolution of the clot. Thereby, BPC 157 deserves to be used in clinical scenario of wound healing external (skin) and/or internal (i.e., abdominal) associated with bleeding disorders. The turmeric agent curcumin has a protective effect on the stomach, but its effectiveness in the lower digestive tract is less known. Zhong et al. found that curcumin is effective in regulating naïve, cell differentiation, TCM and TEM in peripheral blood to ameliorate experimental DSS-induced colitis, and the beneficial effects of this turmeric may depend on inhibition of JAK1/STAT5 signaling activity. Sun et al. studied the role of intestinal glial cells in the mechanism of intestinal barrier disruption in mice with intestinal injury exposed to acute ischemia-reperfusion (I/R). They discovered for the first time that glial cells can modulate intestinal barrier function through A2A adenosine receptors (A2ARs), as a marked exacerbation of I/R damage was observed in A2AR knockout (KO) mice. Moreover, they shed further insight into the mechanism of this protection, demonstrating that A2AR agonists increased barrier proteins ZO-1 and occludin expression in glial cells co-cultured in Caco-2 monolayers, and that the PKCα-dependent signaling pathway in response to hypoxia *in vitro* may be due to a synergistic effect A2AR and metabotropic glutamate receptor 5. It is known that the gaseous mediator’s nitric oxide (NO), hydrogen sulfide (H_2_S) and carbon monoxide (CO) play a key role in the regulation of many physiological functions, including the mechanism of gastrointestinal protection and ulcer healing. Danielak et al. presented an update on the therapeutic properties of a new class of synthesized NSAIDs equipped with NO, H_2_S or CO molecules and releasing one or more of these gaseous messengers. These physiological mediators have attracted widespread attention as agents capable of reducing the side effects of non-steroidal anti-inflammatory drugs (NSAIDs), which are known to be among the most used drugs in the treatment of numerous inflammatory diseases. In their review, the authors cite preclinical and clinical evidence demonstrating the promising anti-inflammatory power and gastrointestinal safety of these new NSAIDs and their mechanisms of action, confirming the real contribution of “gaseous” NSAIDs to the defense mechanism of the gastrointestinal mucosa by reducing the side effects of these parent drugs in the intestine. Honjo et al. provided new insight into the potential target of IBD therapeutic intervention, including receptor-interacting serine/threonine kinase 2 (RIPK2), now also recognized as receptor-interacting protein 2 (RIP2). This small molecule acts as a downstream signaling molecule for nucleotide-binding oligomerization domain 1 (NOD1), NOD2, and Toll-like receptors (TLRs). The biologically active molecule, RIPK2, is expressed in antigen-presenting cells such as dendritic cells and macrophages, triggering the expression and release of pro-inflammatory cytokines such as TNF-α, IL-6 and IL-12/23p40 by activating NFκB and mitogen activated protein kinases, thus playing an important role in infection by microorganisms inducing host defense. This review elucidated the recent advances in RIP2-induced IBD immune-pathogenesis and presented the prospect of therapy for autoimmune disorders such as IBD. Kim and Kim reviewed the physiology and pharmacology of the pluripotent peptide hormone leptin, which is produced by adipocytes but also in the gastric mucosa. Leptin affects the hypothalamus by suppressing appetite and is involved in the regulation of energy homeostasis and neuroendocrine functions. The authors referred to leptin downstream signaling in the digestive tract and accessory digestive organs. The diverse roles of leptin in the digestive system, including immune regulation, cell proliferation, tissue healing and glucose metabolism, are discussed, as well as current therapeutic applications of recombinant leptin. Nicotinamide riboside (NR), vitamin B_3_ and sirtuins play a key role in the aging process, neurodegenerative processes, and myopathy. Hutka et al. provided evidence that ingestion of NR-containing pellets attenuated tumor cachexia and accompanying sarcopenia in a C26 mouse model of adenocarcinoma. In addition, NR administration significantly increased the levels of key enzymes involved in the biosynthesis of NAD^+^ and nicotinamide phosphoribosyl transferase, and significantly inhibited NAD^+^-sensitive sirtuin deacetylase 1 (SIRT1), indicating that NR treatment may be useful in the treatment of human cancer cachexia. Park et al. discussed whether NSAID-induced dysbiosis and associated intestinal disorders are manifested by direct antibacterial effects or delayed peristalsis induced by these drugs. They reported that ketorolac, an NSAID, has a significant effect on the gut microbiota and bile acids in the absence of *mucositis*, and that ketorolac has no effect on gastrointestinal transit delay. Gravina et al. selected a patient population with areas of clarithromycin (CLA) resistance to investigate the efficacy and safety of bismuth quadruple therapy (BQT) or bismuth-free quadruple therapy against *Helicobacter pylori* (Hp) infection. They found that in an area with a high prevalence of CLA-resistant or CLA + metronidazole-resistant Hp strains, the BQT containing Pylera is an effective therapeutic strategy against Hp-infection associated with good adherence and low incidence of adverse events. Park et al. theorized that human placenta-derived mesenchymal stem cells (PD-MSCs) could repair Hp-associated precancerous chronic atrophic gastritis (CAG) in mice, thereby preventing gastric carcinogenicity. The PD-MSC treatment groups showed significantly reduced inflammation, gastric atrophy, erosions/ulcerations, and dysplastic changes and associated with normalization of microbial communities after treatment comparing to pathogenic microbiota in the CAG mice. Nam et al. identified triptolide as a novel NRF2 inhibitor that significantly attenuates ARE-luciferase activity at nanomolar concentrations and reduces NRF2 accumulation in the nucleus, promoting nuclear export of this factor. Moreover, oral administration of triptolide inhibited the growth of A549 xenografts in athymic mice by promoting oxidative damage through nuclear export of NRF2, indicating that inhibition of NRF2 by increasing the cytoplasmic localization of this factor may be the main mechanism of triptolide anti-tumor effect. In a review by Dahlgren et al. the toxicity of chemotherapeutic agents in the gut, leading to chemotherapeutics-induced intestinal mucositis affecting the normal gut microbiota, was described. The authors proposed a combination therapy of chemotherapeutics with prophylactic drugs, such as antibiotics or probiotics, antioxidants, apoptosis inhibitors, anti-inflammatory agents or agents that promote cell proliferation and adaptation. The claudin family, which are transmembrane proteins, are believed to act as the potential targets in patients with IBD, and therefore, Ćužić et al. studied claudin expression in human diseases and two different animal models of IBD: sodium dextran sulfate (DSS)-induced colitis and an adoptive model of transmission of colitis. They found that claudins are not exclusively expressed in epithelial cells, but in some cell types of mesodermal origin, and concluded that these proteins could be considered as a therapeutic target for pharmacotherapy against intestinal inflammation in preclinical animal models and human IBD. Filaretova et al. considered the role of glucocorticoids in the mechanism of distant preconditioning, a therapeutic intervention of short ischemia known to protect organs such as the heart, brain, lungs, and kidneys from various tissue damage because of severe ischemia-reperfusion (I/R). They found that the glucocorticoid synthesis inhibitor metyrapone, which caused a considerable decrease in plasma corticosterone levels, reduced the protective effect of preconditioning in the stomach exposed to I/R, while the corticosterone replacement in rats with adrenalectomy, restored gastric protection exhibited by preconditioning against I/R-induced damage. This is first evidence that glucocorticoids may be involved in the gastroprotective mechanism of ischemic preconditioning against I/R-induced gastric damage. Földes et al. attempted to optimize culture conditions for three-dimensional growth of ameloblast-derived HAT-7 cells useful for assessing the effect of fluoride exposure on HAT-7 spheroid formation. They concluded that such a new 3D model would be suitable for studying the mechanism of amelogenesis, which is evidently affected by exposure to fluoride. Sang-Ngoen et al. conducted a meta-analysis to summarize the effects of probiotics on periodontal disease microbiota and oral health. They described the beneficial effect of probiotics on the reduction of some pathogenic bacterial strains, with little or no effect on others, supporting the notion that, due to the complexity of periodontal disease, there is a need for well-designed randomized clinical trials to evaluate the real effectiveness of probiotics. Nakamura et al. studied the effect of elevated pH induced by acid suppressants, including vonoprazan, lansoprazole and famotidine, on the viability of non-*Helicobacter pylori* helicobacters (NHPH) in mice. They observed that NHPH urease activity as assessed by urea breath tests and the number of these pathogenic bacteria decreased after the use of acid suppressors, i.e., vonoprazan, indicating that NHPH damage may be dependent on changes in pH. Revenko et al. studied the effects of sodium hydrosulfide (NaHS), an H_2_S donor, on stress-induced gastric injury and associated changes in essential H_2_S enzymes, mesenteric vessels, and connective and adipose tissues of aged rats fed a high-fructose diet. They reported that in stressed elderly rats, treatment with NaHS protected mesenteric cell mitochondria and microvascular endothelial and subendothelial structures, including fibroblasts, and restored the activity of H_2_S-related enzymes. They concluded that the H_2_S donor improves both H_2_S signaling and mitochondrial redox balance, which are crucial, especially in advanced age-related high-fructose diet injury. Wang et al. determined the role of metastasis-associated lung adenocarcinoma transcript 1 (MALAT1) in diabetic gastroparesis (DGP). They showed that MALAT1 expression was upregulated in gastric tissues of DGP mice, in adjacent healthy tissues from diabetic gastric cancer patients with symptoms of DGP, and in glucose-fed cultures of human gastric smooth muscle cells. Their study points to a new regulatory signaling pathway in the human diabetic gastroparesis, including MALAT1, miR-449a and delta-like ligand 1. Tepes et al. determined whether the stable gastric pentadecapeptide BPC 157, which has been shown to prevent large vessel occlusion syndromes, may also be effective in a model of multiple occlusion syndrome in rats. By improving the function of the venous system with BPC 157, these authors convincingly demonstrated the counteraction of the chain of arterial-vascular harmful events, the preservation of collateral circulation and the reversal of symptoms not only in gastrointestinal organs such as stomach, but also the cerebral edema and venous and arterial thrombosis, indicating that BPC 157 has cured primary abdominal compartment syndrome. Dong et al. studied the efficacy of naringin in two experimental murine models of colitis *in vivo* and *in vitro* using RAW264.7 cells stimulated with lipopolysaccharide (LPS). They found that this polyphenolic compound alleviated symptoms of colitis and suppressed the LPS-induced high expression of NF-κB-p65, which was further inhibited by small interfering RNAs targeting PPAR-γ, revealing a new therapeutic role for polyphenolic naringin and its usefulness in the treatment of colitis and, perhaps human IBD in the future clinical trials. Skroza et al. presented an analysis of a retrospective single-centre study of the efficacy and safety of isotretinoin in the treatment of moderate to severe acne in real clinical practice. Indeed, early systemic treatment with isotretinoin in patients suffering from moderate acne has shown health benefits, however, the authors recommend an appropriate dose adjustment to minimize side effects. Hou et al. studied the hepatoprotective properties of a natural plant mixture of *Panax notoginseng* (PNM) against mouse liver damage caused by hepatic ischemia/reperfusion (HIR). They documented that PNM exerted a protective effect against HIR-induced hepatic damage and investigated further the plant-pharmacology of the network by evaluating the target genes responsible for this protection by searching various databases that were later validated by immunohistochemical analysis of the mice liver tissues. As a result, they concluded that PNM may exhibit hepatoprotection by reducing the gene expression of nuclear receptor subfamily3 group C member 2 (NR3C2), SRC and GAPDH.

